# Clinical Significance of Low-Flow Time in Patients Undergoing Extracorporeal Cardiopulmonary Resuscitation: Results from the RESCUE Registry

**DOI:** 10.3390/jcm9113588

**Published:** 2020-11-07

**Authors:** Ik Hyun Park, Jeong Hoon Yang, Woo Jin Jang, Woo Jung Chun, Ju Hyeon Oh, Yong Hwan Park, Cheol Woong Yu, Hyun-Joong Kim, Bum Sung Kim, Jin-Ok Jeong, Hyun Jong Lee, Hyeon-Cheol Gwon

**Affiliations:** 1 Division of Cardiology, Samsung Changwon Hospital, Sungkyunkwan University School of Medicine, Changwon 51353, Korea; ionoeval@gmail.com (I.H.P.); saintjmn@naver.com (W.J.C.); ojh@korea.com (J.H.O.); hippomac@hanmail.net (Y.H.P.); 2 Division of Cardiology, Heart Vascular Stroke Institute, Samsung Medical Center, Sungkyunkwan University School of Medicine, Seoul 06351, Korea; jhysmc@gmail.com (J.H.Y.); hc.gwon@samsung.com (H.-C.G.); 3 Division of Cardiology, Department of Internal Medicine, Seoul Hospital, Ewha Womans University College of Medicine, Seoul 07804, Korea; 4 Division of Cardiology, Department of Internal Medicine, Korea University Anam Hospital, Seoul 02841, Korea; ycw717@naver.com; 5 Division of Cardiology, Department of Medicine, Konkuk University Medical Center, Seoul 05030, Korea; drkhj2000@kuh.ac.kr (H.-J.K.); dolphindance98@gmail.com (B.S.K.); 6 Division of Cardiology, Department of Internal Medicine, Chungnam National University Hospital, Daejeon 35015, Korea; jojeong@cnu.ac.kr; 7 Division of Cardiology, Department of Medicine, Sejong General Hospital, Bucheon 14754, Korea; untouchables00@hanmil.net

**Keywords:** extracorporeal cardiopulmonary resuscitation, in-hospital cardiac arrest, low-flow time, vasoactive inotropic score

## Abstract

Limited data are available on the association between low-flow time and survival in patients with in-hospital cardiac arrest (IHCA) who undergo extracorporeal cardiopulmonary resuscitation (ECPR). We evaluated data from 183 IHCA patients who underwent ECPR as a rescue procedure. Patients were divided into two groups: patients undergoing extracorporeal membrane oxygenation as an adjunct to standard cardiopulmonary resuscitation for less than 38 min (*n* = 110) or for longer than 38 min (*n* = 73). The ECPR ≤ 38 min group had a significantly greater incidence of survival to discharge compared to the ECPR > 38 min group (40.0% versus 24.7%, *p* = 0.032). The incidence of good neurologic outcomes at discharge tended to be greater in the ECPR ≤ 38 min group than in the ECPR > 38 min group (35.5% versus 24.7%, *p* = 0.102). The incidences of limb ischemia (*p* = 0.354) and stroke (*p* = 0.805) were similar between the two groups, but major bleeding occurred less frequently in the ECPR ≤ 38 min group compared to the ECPR > 38 min group (*p* = 0.002). Low-flow time ≤ 38 min may reduce the risk of mortality and fatal neurologic damage and could be a measure of optimal management in patients with IHCA.

## 1. Introduction

Several observational studies have shown that extracorporeal cardiopulmonary resuscitation (ECPR) improves survival compared to conventional cardiopulmonary resuscitation (CPR) in patients with cardiac arrest [1,2,3]. ECPR restores indispensable systemic circulation and provides a bridge to possible diagnosis or treatment; therefore, ECPR has the potential to minimize organ damage and prevent re-arrest due to ischemia-triggered myocardial dysfunction [4]. Previous studies demonstrated acceptable survival after ECPR in patients with in-hospital cardiac arrest (IHCA) [1,5] and suggested that the time from arrest to extracorporeal membrane oxygenation (ECMO) might be the primary determinant of successful outcomes [6,7]. Guidelines and expert opinions state that the ideal therapeutic window for ECPR is within 60 min of arrest [8,9,10], and the 60-min cut-off is a commonly used selection criterion at current clinical ECMO centers. However, the evidence for this 60-min cut-off is based on limited data from heterogeneous populations. Moreover, patients rescued by ECPR within 60 min frequently had poor neurologic outcomes. Therefore, we evaluated the association of ECPR time from cardiac arrest to ECMO pump-on with in-hospital mortality and good neurologic status and sought to determine the optimal timing of ECMO insertion during standard CPR in cardiogenic shock patients presenting with IHCA.

## 2. Experimental Section

**Figure 1:**
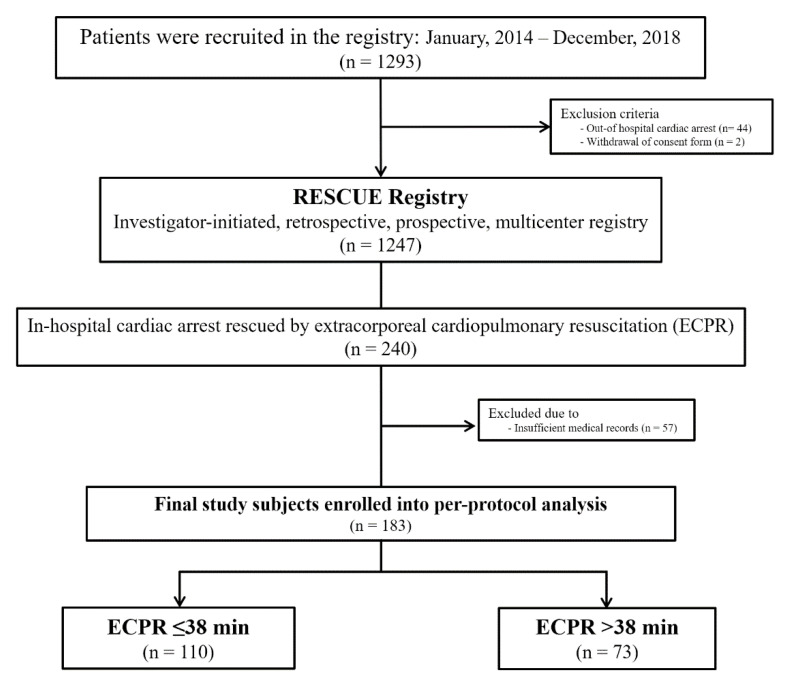
Schematic illustration of study cohort selection.

The RESCUE (REtrospective and prospective observational Study to investigate Clinical oUtcomes and Efficacy of left ventricular assist device for Korean patients with cardiogenic shock; NCT02985008 at www.clinicaltrials.gov) study is a multi-center registry of patients with cardiogenic shock aged over 19 years. Between January 2014 and December 2018, a total of 1247 patients were enrolled from 12 tertiary centers in the Republic of Korea. More detailed information regarding prospective and retrospective enrollments of each institute is shown in Table A1. The inclusion criteria were as follows: (1) systolic blood pressure < 90 mmHg for 30 min or the need for inotrope or vasopressor support to achieve a systolic blood pressure > 90 mmHg and (2) the presence of pulmonary congestion and signs of impaired organ perfusion (altered mental status, cold periphery, oliguria < 0.5 mL/kg/hour for the previous 6 h, or blood lactate > 2 mmol/L). Exclusion criteria were (1) out-of-hospital cardiac arrest and (2) evidence of septic or hypovolemic shock. For this study, we evaluated 240 patients with IHCA rescued by ECPR. Fifty-seven of those patients were excluded due to insufficient medical records. Finally, 183 patients were divided into two groups according to a mean ECPR time of 38 min, the same value as in the risk prediction model of survival to discharge, in which a 38-min ECPR time was defined as an intra-ECPR factor in the prognostic scoring system [11] (Figure 1). The institutional review board of each hospital approved the study protocol and waived the requirement for written informed consent for patients enrolled in this retrospective registry. We obtained informed consent from the patients enrolled in the prospective registry.

Baseline characteristics, procedural characteristics, laboratory data, and clinical outcome data were obtained by reviewing medical records. Laboratory findings, including serum creatinine and lactate, exhibiting the worst values in the 24 h after veno-arterial ECMO insertion were collected. As for recurrent arrest cases, if the duration of return of spontaneous circulation (ROSC) was longer than 20 min, the following arrest event was considered a standard initial point of cardiac massage [1]. Primary outcome was survival to discharge. Good neurologic outcomes at discharge (defined as a Cerebral Performance Category (CPC) score of (1) or (2) [12]), limb ischemia, stroke, and major bleeding were assessed in addition to the primary outcome. Major bleeding was defined hemodynamic instability, requiring transfusion of five red blood cell units over the first day for cannula-related hemorrhage, or injuries that occurred in a critical area or organ such as intracranial, retroperitoneal, pericardial, or intramuscular with compartment syndrome.

The definition of ECPR included both successful veno-arterial ECMO implantation and pump-on during cardiac massage [1,2]. ECPR time was defined as time from initiation of cardiac massage to that of termination of standard CPR due to ECMO operation. CPR was led by the CPR team of the hospital, and all facts related to the CPR scene were recorded according to Utstein style guidelines by bedside nurses [13]. The request call for ECPR was up to the CPR leader and ECMO specialists such as interventional cardiologists or cardiac surgeons made a final decision to initiate ECMO procedure during CPR. The Capiox Emergency Bypass System (Capiox EBS™; Terumo, Inc., Tokyo, Japan) and Permanent Life Support (PLS) System (MAQUET, Rastatt, Germany) were used. After initiation of ECMO, the pump blood flow rate was initially set above 2.2 L/min/body surface area (m^2^) and subsequently adjusted to maintain a mean arterial pressure above 65 mmHg. Blood pressure was continuously monitored through an arterial catheter, and arterial blood gas analysis was performed in the artery of the right arm to estimate cerebral oxygenation. Additional fluids, blood transfusion, and/or catecholamines (i.e., norepinephrine, epinephrine, or dobutamine) were supplied to maintain intravascular volume and/or to achieve a mean arterial pressure above 65 mmHg if necessary [14].

Continuous variables were compared using Student’s t-test or the Wilcoxon rank-sum test and presented as mean ± standard deviation or median (interquartile range, 25th to 75th percentile). Categorical data were tested using Fisher’s exact test or the Chi-square test, as appropriate, and presented as number and relative frequency. Covariates that were either statistically significant on univariable analysis (*p* value < 0.1) or considered clinically important were included in multivariate models. Analyzed covariates were age, body mass index, chronic kidney disease, diabetes mellitus, ECPR time, hypertension, ischemic cardiomyopathy, vasoactive inotropic score (VIS), and shockable rhythm as the first monitored arrest rhythm. All tests were two-tailed, and *p* value < 0.05 was considered statistically significant. Statistical analyses were performed using SPSS software, version 23 (IBM, Armonk, NY, USA).

## 3. Results

### 3.1. Baseline and Arrest Characteristics

Between January 2015 and December 2018, a total of 183 patients received ECPR for IHCA. Baseline, arrest, and resuscitation characteristics of the study population are shown in Table 1. Age tended be older in the ECPR > 38 min group compared to the ECPR ≤ 38 min group (*p* = 0.087), and the incidence of previous myocardial infarction (MI) was significantly higher in the ECPR > 38 min group compared to the ECPR ≤ 38 min group (*p* = 0.045). The most common cause of IHCA was ischemic cardiomyopathy, and almost all patients who had IHCA experienced witnessed arrest and underwent bystander CPR. There were no significant differences in first monitored arrest rhythm, defibrillation, or ROSC before ECMO pump-on between the two groups. However, median total length of stay in the intensive care unit (12.5 days versus 10.0 days, *p* < 0.001) and in the hospital (19.0 days versus 16.0 days, *p* < 0.001) was longer in the ECPR ≤ 38 min group compared to the ECPR > 38 min group.

### 3.2. ECPR and Laboratory Characteristics

ECMO and laboratory characteristics are shown in Table 2. Median duration of ECMO support was 3 days (interquartile range 2.0 to 6.0 days). The operating site of ECMO was different between the two groups (*p* < 0.001), but ECMO was mainly inserted in the catheterization laboratory room (67.8%) and under fluoroscopic guidance (76.5%). Sizes of the femoral arterial and venous cannula were similar between the ECPR ≤ 38 min and ECPR > 38 min groups (*p* = 0.252 and *p* = 0.391, respectively). During ECMO support, anticoagulation therapy, left ventricular venting, distal perfusion, intraaortic balloon pump, continuous renal replacement therapy, and mechanical ventilation were performed similarly in both groups. Laboratory findings just before ECMO insertion including hemoglobin (mg/dL), serum glucose (mg/dL), serum creatinine (mg/dL), serum NT-proBNP (pg/dL), and total bilirubin (mg/dL) did not differ between the ECPR ≤ 38 min and ECPR > 38 min groups. The lactate level before ECMO insertion was greater in the ECPR > 38 min group compared to the ECPR ≤ 38 min group (*p* = 0.010), but lactate level 24 h after ECMO pump-on was not significantly different between the two groups (*p* = 0.172).

### 3.3. Clinical Outcomes

Of the 183 patients in the study population, 101 (55.2%) were successfully weaned from ECMO. The ECPR ≤ 38 min group had a higher incidence of survival to discharge compared to the ECPR > 38 min group (40.0% versus 24.7%, *p* = 0.032). The incidence of good neurologic outcomes at discharge tended to be higher in the ECPR ≤ 38 min group compared to the ECPR > 38 min group (35.5% versus 24.7%, *p* = 0.102). Among the patients who were discharged alive, a lower incidence of good neurologic outcomes was observed in ECPR ≤ 38 min group compared to ECPR > 38 min group, but is was no significant (11.4% versus 0%, *p* = 0.309). The incidences of limb ischemia (*p* = 0.354) and stroke (*p* = 0.805) were not significantly different between the two groups, but major bleeding occurred less frequently in the ECPR ≤ 38 min group than in the ECPR > 38 min group (11.8% versus 30.1%, *p* = 0.002) (Table 3).

### 3.4. Prognostic Factors of Survival to Discharge

**Figure 2:**
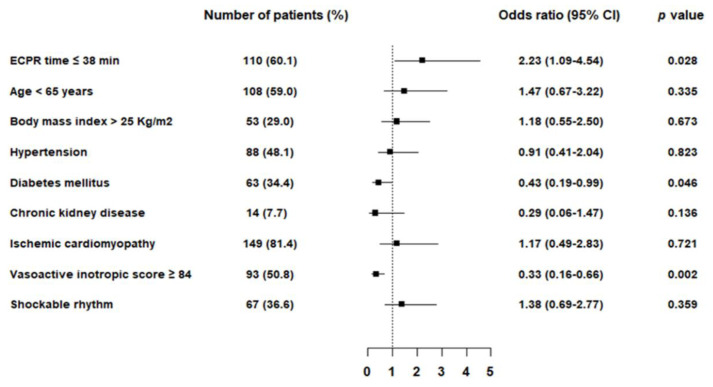
Predictors of survival to discharge. Forest plots show the results of multivariable analysis of predictors of survival to discharge. ECPR = extracorporeal cardiopulmonary resuscitation, CI = confidence interval.

**Figure A1:**
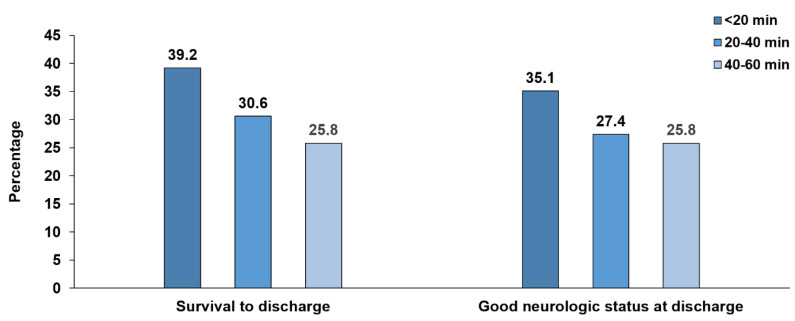
Relation between ECPR duration and clinical outcomes. Bar plots show the relation between ECPR duration and survival to discharge or good neurological outcomes at discharge. ECPR = extracorporeal cardiopulmonary resuscitation.

In multivariable logistic regression analysis, ECPR ≤ 38 min (odds ratio [OR] 2.23; 95% confidence interval [CI] 1.09–4.54; *p* = 0.031) was an independent predictor of favorable survival to discharge. Negative predictors of survival discharge were diabetes mellitus (OR 0.43; 95% CI 0.19–0.99; *p* = 0.046) and VIS ≥ 84 (OR 0.33; 95% CI 0.16–0.66; *p* = 0.002) (Figure 2). The adverse relationship between ECRP duration and survival to discharge or good neurologic outcomes at discharge is shown in Figure A1.

### 3.5. Clinical Outcomes According to Vasoactive Inotropic Score

**Figure 3:**
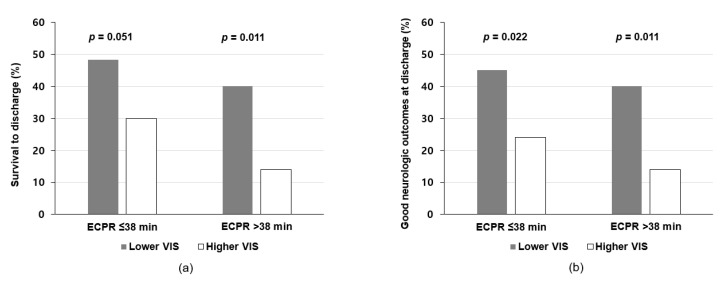
Subgroup analysis of the association between clinical outcomes and VIS or ECPR time. Bar plots show the results of subgroup analysis of the association between clinical outcomes and ECPR time or VIS. (**a**) Survival to discharge, (**b**) Good neurologic outcomes at discharge. ECPR = extracorporeal cardiopulmonary resuscitation, VIS = vasoactive inotropic score.

Subgroup analysis was performed to analyze the relationship between clinical outcomes and VIS or ECPR time. Patients were divided into the lower VIS and higher VIS subgroups according to median VIS (=84) in both ECPR ≤ 38 min and ECPR > 38 min groups. In the ECPR > 38 min group, the incidence of survival to discharge was significantly lower in the higher VIS subgroup than in the lower VIS subgroup (14.0% versus 40.0%, *p* = 0.011). The higher VIS subgroup exhibited a significantly lower incidence of good neurologic outcomes at discharge compared to the lower VIS subgroup in both ECPR time groups, with higher significance observed in the ECPR > 38 min group than in the ECPR ≤ 38 min group (*p* = 0.011 and *p* = 0.022, respectively) (Figure 3).

## 4. Discussion

We investigated the association between time from initiation of standard CPR to ECMO operation and clinical outcomes including survival and neurologic status in cardiogenic shock patients presenting with IHCA treated by ECPR as a rescue procedure. Our main finding is that survival to discharge and good neurologic outcomes at discharge were more frequent in patients with ECPR ≤ 38 min, with fewer bleeding complications noted. Our findings correspond well with earlier studies that established an association between a shorter duration of ECPR and favorable clinical outcomes.

To date, ECMO has been increasingly used under cardiopulmonary collapse without ROSC by standard CPR [15] and several observational studies have shown an improved survival rate with ECPR compared to conventional CPR in patients suffering cardiac arrest [1]. A recently published meta-analysis of IHCA patients rescued by ECPR showed acceptable clinical outcomes with 37.9% of patients surviving to discharge, and good neurological outcomes (CPC 1 or 2) occurring in 84.4% of survivors [5]. IHCA patients treated with ECPR may be affected by pre-ECMO factors (e.g., age, comorbidities, obesity, Glasgow Coma Score, level of lactate, and initial arrest rhythm) [16], intra-ECMO factors (e.g., revascularization, low-flow time, and vascular complications), and post-ECMO factors (e.g., bleeding, limb ischemia, left ventricular distension, and Harlequin syndrome). Among the intra-ECPR factors, ECPR time representative of low-flow time is a clinically unique modifiable factor. Park et al. reported prognostic factors including ECPR time and a prognostic model to help physicians improve survival in IHCA patients and they found that a shorter low-flow time was a predictor of overall survival after ECPR [5,11]. However, they had limited data about in-hospital outcomes and no data regarding the neurologic outcomes of patients. Cho et al. [17] showed that both an experienced ECMO team for priming ECMO circuits and application of ECMO as soon as possible are required to improve survival after cardiac arrest and suggested that CPR time > 12 min is a unique prognostic factor for in-hospital mortality in patients treated with ECMO-associated primary PCI for ST-segment elevation MI (STEMI). However, they evaluated limited data only from patients with cardiac arrest complicating STEMI; moreover, ECPR less than 12 min is impossible to apply in clinical practice, and the rationale for that criterion remains unclear. We evaluated a prognostic value for neurologic outcomes as well as survival to discharge using a large multicenter dedicated shock registry including diverse cardiac problems and demonstrated that patients with ECPR ≤ 38 min, which is practical and rational criterion for clinical application, had survival benefits compared with patients with ECPR > 38 min. As exemplified by a previous study indicating that the probability of survival to discharge decreased to 0.45, 0.37, 0.30, 0.24, and 0.18 as the ECMO pump-on time was delayed to 20, 30, 40, 50, and 60 min, respectively, it seems obvious that further reducing duration of CPR to ECMO pump-on time would improve long-term prognosis [11,15]. In terms of the short and feasible time criterion, ECPR ≤ 38 min could be an indicator of lower mortality and good neurologic status and, in particular, it may aid in determination of optimal timing from standard CPR to ECMO application in IHCA patients and prevent ECMO misuse or overuse in IHCA patients with recovery of spontaneous cardiac function. Therefore, it may be necessary to establish a hospital-specific strategy for ECMO pump-on within an acceptable low-flow time, with availability to be easily performed in current clinical practice. Our findings could provide relevant insight into early and long-term mortality and neurologic prognosis in patients treated with ECPR for IHCA in real-world practice.

IHCA patients treated with ECPR often exhibit fatal arrhythmias and multiple organ dysfunction, which catecholamines can aggravate by inducing tachy-arrhythmia and increasing oxygen consumption, complicating recovery after ROSC [18]. We investigated the positive or negative predictors of survival discharge for IHCA patients who underwent ECPR and our multivariable analysis showed that not only ECPR ≤ 38 min but also VIS ≥ 84 were powerful predictors of survival to discharge. This result is in accordance with a previous study that showed a significant association between higher levels of vasoactive inotropic support and increased in-hospital mortality in patients with cardiogenic shock regardless of ECMO support [19]. Moreover, in subgroup analysis, worse neurologic outcomes at discharge as well as survival to discharge were obtained in patients undergoing longer ECPR time with higher VIS. A neuroprotective effect of ECMO in patients treated with CPR was observed in a previous study showing that prolonged interval from cardiac arrest to ECMO operation was associated with poor neurologic outcomes after ECPR [20]. In addition, vasopressors can negatively affect cerebral oxygenation, leading to a detrimental effect on neurologic outcomes [21]. Given that patients with severe cardiogenic shock associated with impairment of end-perfusion organs had the worst prognosis [22,23], based on our results, excessive administration of vasopressors after ECPR could worsen neurologic prognosis as well as survival rate, especially in patients receiving prolonged ECPR. Our results suggest that both careful determination of vasopressor or inotrope dosage and optimization of CPR to ECMO pump-on time should be taken into consideration when ECPR is performed in IHCA patients. Once ECMO was initiated, to avoid excessive administration of vasopressors, adjustment of pump blood flow rate could be considered first rather than increasing vasopressor support to maintain the target arterial pressure.

This study has several limitations. First, its design was non-randomized and observational, potentially affecting the results due to selection bias and confounding factors. Most patients presented with an ischemic etiology, and patients with non-ischemic causes were heterogeneous and of limited sample size. Second, our registry did not include hemodynamic parameters such as pulmonary capillary wedge pressure measured by a pulmonary arterial catheter. Third, treatment of cardiac arrest such as type and amount of fluids and vasopressor/inotropes administered or type and timing of ECMO were left to the physician’s discretion, although coronary intervention was performed based on the guidelines of the Korean Circulation Society. Fourth, due to the retrospective as well as prospective nature of our registry, 23.7% patients were excluded due to insufficient medical records and we could not thoroughly identify the detailed data on clinical outcomes such as minor bleeding, and number of transfused units and procedural characteristics including transition time from CPR to ECPR, and rate of ECPR preceding PCI, which may have limited our results. Fifth, this study included patients with only cardiogenic shock undergoing ECPR and all the patients experienced witnessed arrest and underwent bystander CPR. Therefore, when assessing ECPR in patients with cardiogenic shock accompanied by cardiac arrest, it can be difficult to distinguish the true impact of ECPR alone.

## 5. Conclusions

In patients with cardiogenic shock accompanied by IHCA, lower in-hospital mortality and more favorable neurologic status were observed in patients with a CPR to ECMO pump-on time ≤ 38 min. Based on our results, a fast transition from standard CPR to ECMO operation within low-flow time less than 38 min could reduce mortality and improve neurologic outcomes.

## Figures and Tables

**Table 1:** Baseline, arrest, and resuscitation characteristics.

	Overall Population	ECPR ≤ 38 min	ECPR > 38 min	*p* Value
	*n* = 183	*n* = 110	*n* = 73	
Age (years)	61.6 ± 12.8	62.9 ± 12.4	59.6 ± 13.1	0.087
Gender (male)	135 (73.8)	78 (70.9)	57 (78.1)	0.280
Body mass index (kg/m^2^)	23.6 ± 3.4	23.7 ± 3.6	23.6 ± 3.0	0.806
Medical history				
Hypertension	88 (48.1)	55 (50.0)	33 (45.2)	0.525
Diabetes mellitus	63 (34.4)	43 (39.1)	20 (27.4)	0.103
Dyslipidemia	44 (24.0)	26 (23.6)	18 (24.7)	0.874
Current smoker	62 (33.9)	35 (31.8)	27 (37.0)	0.469
Chronic kidney disease	14 (7.7)	11 (10.0)	3 (4.1)	0.142
Peripheral vascular disease	12 (6.6)	9 (8.2)	3 (4.1)	0.276
Previous MI	26 (14.2)	11 (10.0)	15 (20.5)	0.045
Previous PCI	31 (16.9)	18 (16.4)	13 (17.8)	0.799
Previous CABG	5 (2.7)	4 (3.6)	1 (1.4)	0.357
Previous CVA	19 (10.4)	10 (9.1)	9 (12.3)	0.482
Left ventricular ejection fraction (%)	29.6 ± 17.1	29.8 ± 16.3	29.3 ± 18.3	0.860
Use of Vasopressor or Inotropic	172 (94.0)	104 (94.5)	68 (93.2)	0.697
Vasoactive inotropic score	84.0 (22.0–199.0)	59.5 (20.0–173.0)	99 (40.0–200.0)	0.178
Purpose of ECMO implantation				
Bridge to recovery	80 (43.7)	44 (40.0)	36 (49.3)	0.214
Bridge to revascularization	32 (17.5)	23 (20.9)	9 (12.3)	0.135
Bridge to heart transplantation	2 (1.1)	1 (0.9)	1 (1.4)	0.769
Bridge to decision	74 (40.4)	46 (41.8)	28 (38.4)	0.640
Clinical presentation				0.598
Ischemic cardiomyopathy	151 (82.5)	90 (81.8)	61 (83.6)	
Dilated cardiomyopathy	1 (0.5)	0 (0)	1 (1.4)	
Fulminant myocarditis	4 (2.2)	4 (3.6)	0 (0)	
Valvular heart disease	6 (3.3)	4 (3.6)	2 (2.7)	
Refractory arrhythmia	8 (4.4)	5 (4.5)	3 (4.1)	
Massive PTE	7 (3.8)	4 (3.6)	3 (4.1)	
Other causes	6 (3.3)	3 (2.7)	3 (4.1)	
First monitored arrest rhythm				0.847
Asystole	52 (28.4)	31 (28.2)	21 (28.8)	
Pulseless electrical activity	64 (35.0)	37 (33.6)	27 (37.0)	
Shockable rhythm (VF or VT)	67 (36.6)	42 (38.2)	25 (34.2)	
Witnessed cardiac arrest	183 (100.0)	110 (100.0)	73 (100.0)	
Bystander-performed CPR	183 (100.0)	110 (100.0)	73 (100.0)	
Defibrillation	97 (53.0)	57 (51.8)	40 (54.8)	0.693
ROSC before ECMO pump-on	66 (36.1)	36 (32.7)	30 (41.1)	0.248
Length of ICU stay (day)	6.0 (2.0–13.0)	12.5 (6.0–19.0)	10 (5.0–12.0)	<0.001
Length of hospital stay (day)	9.0 (3.0–23.0)	19.0 (10.0–33.0)	16.0 (7.0–30.0)	<0.001

Data are shown as n (%) or median (interquartile range). CABG = coronary artery bypass grafting, CPR = cardiopulmonary resuscitation, CVA = cerebrovascular accident, ECMO = extracorporeal membrane, oxygenation, ECPR = extracorporeal cardiopulmonary resuscitation, ICU = intensive care unit, MI = myocardial infarction, PCI = percutaneous coronary intervention, PTE = pulmonary thromboembolism, ROSC = return of spontaneous circulation, VF = ventricular fibrillation, VT = ventricular tachycardia.

**Table 2:** ECMO and laboratory characteristics.

	Overall Population	ECPR ≤ 38 min	ECPR > 38 min	*p* Value
	*n* = 183	*n* = 110	*n* = 73	
Operating site of ECMO				0.001
Catheterization laboratory room	124 (67.8)	82 (74.5)	42 (57.5)	
Emergency room	23 (12.6)	10 (9.1)	13 (17.8)	
Intensive care unit	25 (13.7)	8 (7.3)	17 (23.3)	
Operating room	11 (6.0)	10 (9.1)	1 (1.4)	
Fluoroscopic guidance	140 (76.5)	88 (80.0)	52 (71.2)	0.171
Arterial cannula size (Fr.)	16.0 ± 0.9	15.9 ± 0.9	16.1 ± 0.8	0.252
Venous cannula size (Fr.)	21.0 ± 1.6	20.9 ± 1.6	21.2 ± 1.5	0.391
Initial ECMO pump flow (L/min)	2.9 ± 1.0	2.9 ± 0.9	3.0 ± 1.1	0.370
During ECMO support				
Anticoagulation therapy	164 (89.6)	98 (89.1)	66 (90.4)	0.774
Left ventricular venting	4 (2.1)	3 (2.7)	1 (1.4)	0.173
Distal perfusion	63 (34.4)	36 (32.7)	27 (37.0)	0.553
Intraaortic balloon pump	29 (15.8)	17 (15.5)	12 (16.4)	0.858
Continuous renal replacement therapy	75 (41.0)	45 (40.9)	30 (41.1)	0.980
Mechanical ventilation	171 (93.4)	100 (90.9)	71 (97.3)	0.089
Duration of ECMO support (day)	3.0 (2.0–6.0)	3.0 (2.0–6.0)	3.0 (2.0–5.0)	0.490
Laboratory findings (just before ECMO insertion)				
Hemoglobin (mg/dL)	12.8 ± 3.2	12.7 ± 3.2	12.9 ± 3.1	0.613
Serum glucose (mg/dL)	256.1 ± 128.4	249.2 ± 123.8	266.3 ± 135.1	0.390
Creatinine (mg/dL)		1.5 ± 1.5	1.4 ± 1.1	0.668
NT-proBNP (pg/mL)	2461.0 (217.0–8150.5)	2461.0 (454.0–7935.5)	1660.0 (133.0–7397.0)	0.599
Total bilirubin (mg/dL)	1.0 (0.0–1.0)	1.0 (0.0–1.0)	1.0 (0.0–1.0)	0.436
Lactate level (mmol/L)				
Just before ECMO insertion	9.6 ± 4.9	8.8 ± 4.7	10.8 ± 5.0	0.010
24 h after ECMO insertion	0.7 ± 0.5	0.6 ± 0.5	0.7 ± 0.5	0.172

Data are shown as *n* (%) or median (interquartile range). ECMO = extracorporeal membrane oxygenation, ECPR = extracorporeal cardiopulmonary resuscitation, NT-proBNP = N-terminal pro b-type natriuretic peptide.

**Table 3:** Clinical outcomes and complications.

	Overall Population	ECPR ≤ 38 min	ECPR > 38 min	*p* Value
	*n* = 183	*n* = 110	*n* = 73	
Survival to discharge	62 (33.9)	44 (40.0)	18 (24.7)	0.032
* Good neurologic outcomes at discharge	57 (31.1)	39 (35.5)	18 (24.7)	0.102
Limb ischemia	17 (9.3)	12 (10.9)	5 (6.8)	0.354
Stroke	11 (6.0)	7 (6.4)	4 (5.5)	0.805
Major bleeding	35 (19.1)	13 (11.8)	22 (30.1)	0.002

Values are *n* (%). * Good neurologic recovery or favorable neurologic status was defined as a cerebral performance category score of 1 or 2. ECPR = extracorporeal cardiopulmonary resuscitation.

## Appendix A

**Table A1:** Prospective and Retrospective Enrollments of each Institute.

Institutes	Overall Population	Retrospective	Prospective
	(*n* = 1247)	(*n* = 954)	(*n* = 293)
Samsung Medical Center	249	144	105
Severance Cardiovascular Hospital	181	147	34
Korea University Anam Hospital	134	130	4
Samsung Changwon Hospital	122	46	76
Konkuk University Hospital	112	89	23
Chungbuk National University Hospital	91	90	1
Inje University Ilsan Paik Hospital	78	64	14
Sejong General Hospital	66	60	6
Chung-Ang University Hospital	67	63	4
Chungnam National University Hospital	57	57	0
Inha University Hospital	52	32	20
Dankook University Hospital	38	32	6
